# T-RAIM for Precise Orbit Determination in LEO-PNT

**DOI:** 10.3390/s25237322

**Published:** 2025-12-02

**Authors:** Ciro Gioia, Francesco Menzione, Andrea Piccolo, Stefano Casotto, Massimo Bardella

**Affiliations:** 1 European Commission, Joint Research Centre (JRC), 21027 Ispra, Italy; ciro.gioia@ec.europa.eu (C.G.); andrea.piccolo@ec.europa.eu (A.P.); 2 Department of Physics and Astronomy, University of Padua, 35122 Padova, Italy; stefano.casotto@unipd.it (S.C.); massimo.bardella@unipd.it (M.B.)

**Keywords:** LEO-PNT, P2OD, T-RAIM, ODTS, Hardware-in-the-Loop

## Abstract

The rapid development of Low Earth Orbit Position, Navigation, and Timing (LEO-PNT) constellations presents opportunities to augment Global Navigation Satellite Systems (GNSSs) with additional signals from Low Earth Orbit (LEO) satellites, thereby improving performance and reliability for users. This research study addresses the challenges posed by the interdependency between LEO and GNSS layers, which can lead to cascading faults. By extending Receiver Autonomous Integrity Monitoring (RAIM)-like capabilities to spaceborne receivers, specifically through Timing Receiver Autonomous Integrity Monitoring (T-RAIM), this paper aims to mitigate these risks. This study validates the integration of T-RAIM with advanced Precise Real-Time On-board Orbit Determination (P2OD) techniques in LEO scenarios using a hardware-in-the-loop test environment. The findings demonstrate that the architecture with T-RAIM can maintain nominal positioning and timing accuracy even in the presence of GNSS clock faults, ensuring continuous system functionality without requiring P2OD restarts. This capability is crucial to preventing service interruptions and enhancing the robustness of LEO-PNT solutions. The proposed integration handles the computational load and complexity while accommodating the limited resources of spaceborne receivers, offering a viable and robust LEO-PNT solution. The experimental results show that T-RAIM effectively mitigates the impact of pseudorange ramp errors, maintaining stable clock bias and preserving the integrity of orbit determination and time synchronization.

## 1. Introduction

Space-based Position, Navigation, and Timing (PNT), with a GNSS as its core, is rapidly evolving. This evolution is driven by emerging LEO-PNT solutions that utilize diverse architectures and new business models [1]. Europe is actively participating in this evolution through initiatives like the European Space Agency (ESA) LEO-PNT project [2] and proposals to integrate PNT capabilities into existing and future satellite communication systems, such as Infrastructure for Resilience, Interconnectivity and Security by Satellite (IRIS2) [3]. In the first case, dedicated LEO constellations [4] are being developed to complement the GNSS infrastructure with innovative LEO-PNT technologies, targeting deployment of demonstrators by 2026. The second initiative aims to repurpose 5G-Non-Terrestrial Network (NTN) LEO constellations through a hybrid approach [5], providing users with additional signals from an extensive network of LEO satellites. Regardless of the chosen approach, all LEO-PNT initiatives aim to deliver cost-effective solutions through multi-tiered architectural designs [6]. In such architectures, LEO-PNT services rely on GNSS receivers hosted on LEO spacecraft, functioning as autonomous systems for on-board Orbit Determination and Time Synchronization (ODTS). These systems generate ephemeris and other necessary corrections that are distributed to LEO-PNT end users, taking over conventional ground-based tasks. The driving technology behind this advancement is the innovative P2OD capability to provide real-time decimeter-level spacecraft orbit reconstruction and, crucially, unbiased nanosecond-level time synchronization. The P2OD algorithm is a sophisticated tool essential to accurately determining satellite orbits by using on-board data from a spaceborne GNSS receiver; the traditional P2OD algorithms do not include integrity checks given the mission-critical requirements of conventional missions. The exploitation of P2OD in LEO-PNT systems completely changes the scenario, shifting it from a mission-critical to a safety-critical component.

Due to the inherent complexity and significant computational demands of the P2OD approach, it is essential to streamline the process by incorporating an integrity check as a preliminary step. The integrity check plays a vital role in the preprocessing phase by identifying and removing outliers from the dataset that will be utilized by the P2OD algorithm. By eliminating the outliers early on, the integrity check ensures that only error-free measurements are fed into the P2OD block. This preprocessing step significantly improves the accuracy and reliability of the resulting orbital parameters.

The issue of integrity for LEO-PNT has been previously discussed in [7], with authors highlighting its importance, especially for users operating in safety-critical navigation sectors. Recent research has further detailed the specific challenges and solutions for LEO-PNT integrity. Although [6] mentions extension of RAIM as a standard method used in the extended LEO plus GNSS domain, specific details regarding LEO-PNT applications are now available through numerical simulations. For instance, the paper by [8] analyzes potential dependencies between Global Positioning System (GPS) and LEO-PNT constellations and their impact on integrity. They show that neglecting the correlation (which arises from the LEO system’s use of GNSSs for ODTS) can adversely affect the calculated Protection Levels (PLs), using solution separation as the evaluation method. Further contributing to this field, the work by [9] analyzed GNSS Augmentation by LEO satellites, specifically focusing on Integrity Performance Under Non-Ideal Conditions. The concept of leveraging the LEO layer for integrity monitoring is gaining traction, with [10] specifically investigating integrity monitoring and augmentation of GNSSs from LEO constellations as a method for monitoring from space.

The critical issue of integrating integrity capabilities into the LEO-PNT ODTS process has been initially introduced in [11]. Similarly, ref. [12] anticipates the need for a safe integration of LEO constellations with GNSSs, emphasizing the necessity of effective integrity monitoring to mitigate cascading effects. Furthermore, the paper in [13] underscores the vital need to complement ground monitoring by incorporating this capability into spaceborne receivers to enhance the robustness of LEO-PNT applications. Despite the challenges posed by on-board RAIM in LEO-PNT, particularly concerning positioning, our research strategically focuses on timing. On the user segment side within the LEO-PNT multi-tier architecture, while ephemeris errors can be mitigated by projecting the most recent valid ephemeris, thereby offering users a grace period, timing errors present a greater challenge, as they directly impact the generation of LEO-PNT signals, leading to errors in the pseudoranges and Carrier-Phase measurements. With respect to the available literature, this paper makes three fundamental contributions:

- It explores and validates T-RAIM algorithms in situations characterized by high dynamics and rapid visibility changes. These conditions are prevalent in LEO settings, where satellites move swiftly relative to the observer, leading to frequent and sudden changes in signal visibility. This study aims to demonstrate the robustness and reliability of T-RAIM algorithms under such challenging conditions, which have not been extensively explored in the existing literature.
- It provides an in-depth analysis of an open-loop integration architecture that combines T-RAIM with P2OD within the LEO-PNT framework. This approach ensures real-time processing and quick detection of the outliers, crucial in LEO environments
- It demonstrates the proposed architecture using the Joint Research Centre (JRC) test environment [14]. In particular, hardware-in-the-loop testing is used for demonstration.

The remainder of the paper is structured as follows: Section 2 details the P2OD and T-RAIM algorithms, Section 3 describes the experimental setup, Section 4 discusses the results, Section 5 discusses the benefits and limitations of the proposed approach, and Section 6 concludes the paper.

## 2. LEO-PNT ODTS Function Integrating T-RAIM

**Figure 1:**
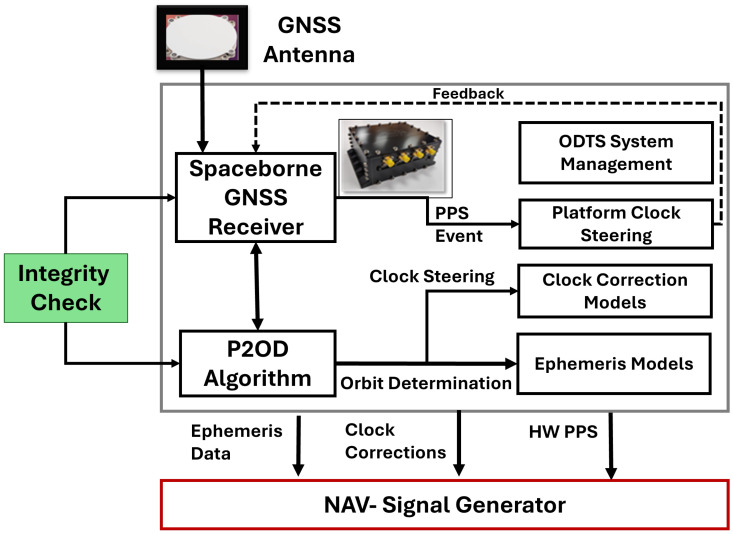
LEO-PNT ODTS architecture.

The general payload architecture for implementing a multi-tier approach is depicted in Figure 1. A dual- or triple-frequency spaceborne GNSS receiver supplies data to the P2OD engine, which generates precise estimates of orbital position and clock bias and drift. This allows for accurate alignment with the target timing reference provided by the backbone GNSS infrastructure. Dedicated processing blocks on board process this information to create a corresponding navigation message, similar to those provided by P2OD, enabling end users to precisely reconstruct the satellite position and correct raw measurements. The clock bias estimation error is targeted to be maintained at the nanosecond level and is used to adjust the GNSS spaceborne Pulse Per Second (PPS), ensuring clock stability and disciplining the transmitter that generates the navigation signals. This architectural design creates significant interdependence between Medium Earth Orbit (MEO) and LEO constellations, suggesting that any malfunctions in the MEO layer can propagate to the LEO layer, potentially causing a domino effect that negatively impacts end-user performance. In this study, an integrity check to filter out faulty measurements before executing P2OD is introduced, as shown in Figure 1. The Fault Detection and Exclusion (FDE) process is carried out outside the P2OD core by a T-RAIM module to decrease the computational burden. The execution of P2OD should occur only after all necessary recursive positioning iterations for satellite exclusion have been completed, aligning with the resource constraints discussed in [4].

### 2.1. Pre-Filtering T-RAIM

The building blocks of the integrity algorithm developed for this study are presented below.

#### 2.1.1. Global Test

To detect a blunder within the measurements, a statistical test can be performed on the residuals and is usually called Global Test (GT). This test verifies the consistency of the measurement set [15]. The GT is based on the null hypothesis that the adjustment model is correct and the distributional assumptions meet reality, with errors assumed to be Gaussian with zero mean. The alternative hypothesis assumes that the adjustment model is not correct. The statistical variable used to test the null hypothesis is the quadratic form of the residual, weighted by the weighting matrix. A common procedure consists of fixing the probability of false alarm, typically set to 0.1% [16], and letting the threshold vary with the redundancy. If the null hypothesis is rejected, an inconsistency in the measurement set is assumed, and the blunder should be identified and mitigated; the identification of the erroneous measurement is carried out by the Local Test (LT).

#### 2.1.2. Local Test

To identify an outlier, an individual measurement test, known as the LT, is performed; it uses the standardized residuals, defined as the absolute value of the residual divided by the square root of the corresponding diagonal element of the residual covariance matrix. The largest standardized residual exceeding the threshold is flagged as a blunder, and it is rejected [17,18].

#### 2.1.3. SLOPE and TPL

The robustness of the system to support the integrity checks is a fundamental element of the RAIM algorithms. Traditional approaches verify the integrity robustness using positioning related parameters such as the Approximate Radial-error Protection (ARP) and the Horizontal Protection Level (HPL), as described in [19]. This study specifically focuses on timing, and Weighted Approximated Time Protected (WATP) and Time Protection Level (TPL) are derived for the time aspect [20]. The TPL is composed of two elements:

- WATP, containing information on the measurement biases;
- The TPL noise term, representing the measurement noise.

WATP is calculated based on the Weighted Timing Slope (WTS), which is a weighted version of the Timing Slope (TS). The WTS is computed as

(1) $$WTS_{i}=\frac{A_{4,i}}{S_{i,i}}$$

WTS is a vector containing the WTS values for all the satellites used in the navigation solution, and the index *i* identifies the satellite. The matrix *A* is computed as

(2) $$A={\left(H^{T}\cdot W\cdot H\right)}^{-1}\cdot H\cdot W$$

where *H* is the design matrix [21] and *W* is the weighting matrix. The weighting model is based on satellite elevation, and the weighting matrix is structured as diagonal. Additionally, an a priori sigma of 1.5 m is adopted for the code measurements. *S* is the slope matrix, and it is computed as

(3) S=I−H·A

with *I* being the identity matrix. The maximum value of the vector WTS is used to compute WATP:

(4) $$WATP=maximum\left(WTS\right)\cdot p_{bias}$$

The SLOPE parameter is defined as the ratio of the estimation error (horizontal, vertical, or timing, as in this study) to the test statistic [22], calculated under the assumption that a deterministic error is present in a single measurement while ignoring any stochastic perturbations [19]. The satellite causing the largest estimation error due to its bias error is the most challenging to detect and is linked to the maximum SLOPE value, denoted by SLOPE max.

#### 2.1.4. Forward/Backward Scheme

**Figure 2:**
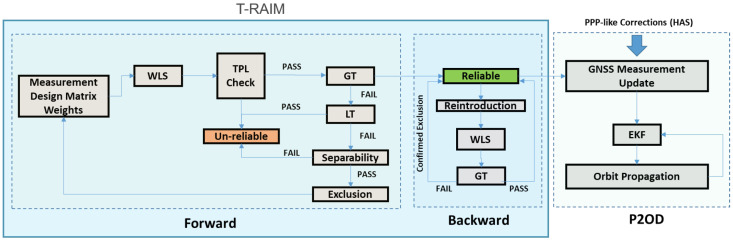
Flowchart of the Forward/Backward algorithm for integrity monitoring in LEO-PNT systems.

The tests presented above can be combined in different ways, leading to different FDE schemes. One of the simplest is obtained using only the GT and, in case of failure testing, all the possible subset combinations of the measurements to identify a unique subset not containing the outlier. Although this approach is conceptually simple, it requires a large computational effort. In this study, we adopted the Forward/Backward scheme [15] specifically tuned for timing application, as described in [20]. The scheme of the developed algorithm and the interaction with the P2OD solution are shown in Figure 2. The flowchart in Figure 2 provides a visual representation of the Forward/Backward algorithm used for integrity monitoring in LEO-PNT systems. It illustrates the sequence of steps involved in the iterative process to ensure reliable satellite measurements. On the left side of the figure, the initial steps are represented and constitute the T-RAIM block, which ensures the check of the measurements to identify and exclude the outliers. The process concludes when the measurement set is deemed reliable, ensuring robust position determination in dynamic LEO environments, and the measurement set without the outlier is passed to the P2OD block.

The algorithm operates in two main phases: the forward phase and the backward phase:

1. Forward Phase:
  - Geometry Check: This initial step involves assessing the robustness of the satellite geometry using the TPL, which must be smaller than a predefined threshold known as the Time Alarm Limit (TAL) to ensure that the system’s geometry is adequate for integrity checks.
  - Once the geometry is confirmed as adequate, the algorithm performs the GT.
  - If the GT detects an inconsistency, the LT is applied to pinpoint the specific measurement.
  - Before excluding a measurement identified by the LT as an outlier, a separability check is performed. This step ensures that the identified outlier is not highly correlated with other measurements, which could make it difficult to isolate genuine errors. If the measurement passes the separability check, it is excluded from the solution.
2. Backward Phase: This phase is triggered if multiple exclusions occurred during the forward phase. The backward phase aims to reintroduce any measurements that might have been erroneously excluded. This is performed using only the GT, without further individual tests, to see if the overall measurement set becomes consistent with all or some of the previously excluded measurements reintroduced.
  The algorithm is designed to declare the solution unreliable under three circumstances:
  - Inadequate Geometry, if the satellite geometry is not robust enough to support the integrity checks.
  - Inconsistency Detection, if the GT indicates an inconsistency but the LT does not identify any specific outliers.
  - High Correlation: If the LT identifies an outlier but it is too closely correlated with other measurements, it may not be possible to accurately identify the true source of error.

The Forward/Backward mechanism is an iterative process; the forward phase terminates under several conditions: if the satellite geometry is found inadequate, if the GT indicates inconsistency but the LT fails to pinpoint specific outliers, if the LT identifies an outlier that is too closely correlated with other measurements, or if no more outliers are identified. Once the forward phase completes, the backward phase is triggered if multiple exclusions occurred. This phase aims to reintroduce any erroneously excluded measurements by using only the GT, without further individual tests, to achieve a consistent overall measurement set. The process ultimately terminates when the measurement set is deemed reliable.

### 2.2. P2OD Algorithm

At the core of P2OD algorithms is a reduced dynamic approach [23] that accounts for various forces acting upon a satellite, including gravitational forces from the Earth, the Moon, the Sun, and other celestial bodies, as well as non-gravitational forces, like atmospheric drag, solar radiation pressure, and relativistic effects. The joint estimation of the orbital state vector and dynamic model parameters is possible via real-time sequential estimation techniques like Kalman filtering, which properly accommodate uncertainties in both measurement data and dynamical models based on spaceborne receiver raw-measurement processing. In this work, the real-time processing for orbit determination of a LEO satellite is performed using a P2OD Software (SW) tool developed by University of Padua (UniPD) [24,25]. This processing employs an Extended Kalman Filter (EKF) algorithm with a reduced-dynamics approach to estimate the complete satellite dynamical state vector, ensuring solution continuity through orbit propagation. The extended Kalman is the baseline approach considered to cope with the target on-board processing and the expected reduced resources of spaceborne receivers. Table 1 includes the key elements of the P2OD algorithm: force models, observation model, numerical integration, and estimation. The force models consider the Earth gravity field modeled with EIGEN-6c4 [26], third-body perturbations from the Sun and Moon using a Point Mass model, and corrections for solid Earth tides as per IERS 2003 standards [27]. Relativity is addressed with post-Newtonian corrections, while solar radiation pressure and atmospheric drag are modeled using a cannon-ball approach, with the latter using the NRLMSISE-00 density model [28]. Empirical accelerations are modeled as a first-order Gauss–Markov process. The observation model leverages ionosphere-free combinations of dual-frequency pseudorange and Carrier Phase, utilizing frequencies E1−E5a for Galileo and L1−L5 for GPS. The software operates in multi-constellation mode. Numerical integration is executed using the Runge–Kutta–Hull method [29].

The estimation process determines state parameters such as position, velocity, drag and solar radiation pressure coefficients; empirical accelerations; receiver clock error; inter-system bias; and Carrier-Phase ambiguities. Pseudorange (PR) measurement noise is set to 4 cm, while Carrier-Phase (CP) measurement noise is set to 2 mm. The float ambiguity process noise is maintained at the millimeter level.

## 3. Hardware-in-the-Loop Testing

This section outlines the hardware-in-the-loop test environment JRC Integrated SSV Test Bench (JRC-ISSVTB) [14]. The test setup integrates hardware and software components, including modules for input data management, high-fidelity spacecraft orbit and attitude propagation, and simulator. The experimental setup supports various mission profiles and vehicle dynamics.

The simulation takes into account two GNSSs: GPS and Galileo. For Galileo satellites, signals were simulated on the E1 and E5a frequencies, while for GPS, signals were simulated on the L1 and L5 frequencies. This approach creates a dual-constellation, dual-frequency scenario, which is the current baseline scenario for P2OD [23]. For the LEO-PNT use case, a single LEO satellite serves as a representative of the target constellation, following a nearly circular, non-Sun-synchronous trajectory at a mean altitude of 1336 km [25]. The scenario duration is about 22 h, with updates to the reference orbit every 100 milliseconds. This setup allows for the comprehensive testing of navigation and timing performance. The fault injection process in this test environment is designed to simulate pseudorange errors arising from clock issues in GNSS satellites, allowing for the rigorous testing of fault detection algorithms. This is achieved by introducing controlled pseudorange ramps to selected satellites, with faults injected based on the SLOPE parameter, which identifies the satellite whose bias error results in the greatest position error. In this study, pseudorange errors are implemented in four satellites per constellation, with each ramp having a maximum pseudorange offset of approximately 500 m, a ramp-up time of ten minutes, and a hold time of one hour, as detailed in Table 2. The values of the ramp are set according to Ref. [30]. The fault injection timing varies according to satellite availability, ensuring that the scenario includes multiple simultaneous faults, thereby providing a challenging environment to test the robustness and effectiveness of the fault detection mechanisms against different satellite geometries and simultaneous anomalies.

## 4. Results

This section presents the experimental results; at first the results of P2OD without the T-RAIM algorithm are shown. The performance of the T-RAIM algorithm is analyzed, and finally, the results of P2OD including pre-filtering are discussed. The performance is evaluated in terms of orbit error computed with respect to the reference trajectory used to feed the radio frequency simulator. Statistical parameters such as mean with standard deviation for each component are evaluated. The performance of T-RAIM is evaluated by analyzing the capability of the algorithm to identify and exclude faulty satellites.

### 4.1. T-RAIM Algorithm Results

**Figure 3:**
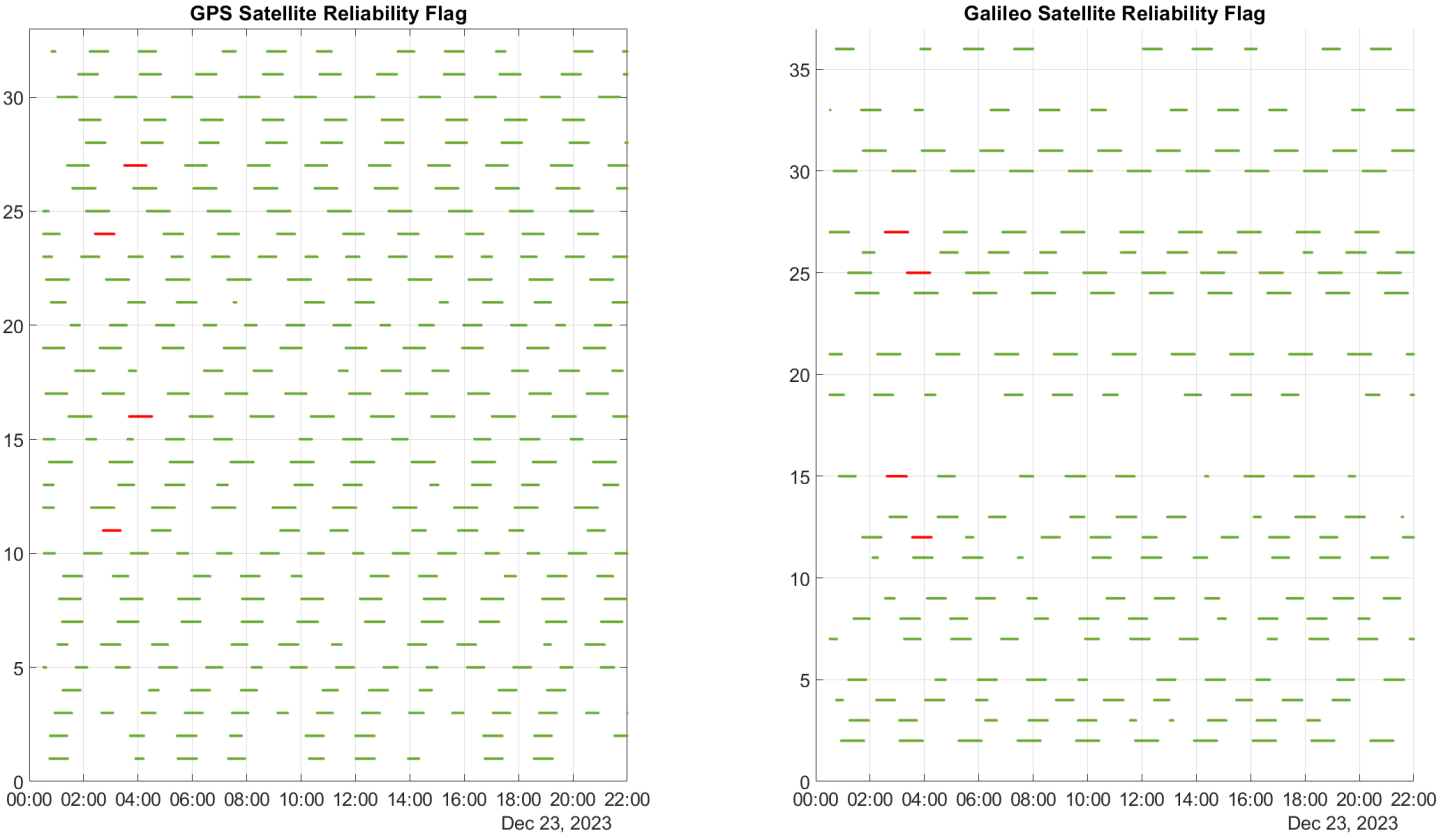
Reliability flag for GPS and Galileo constellations, where green markers indicate that the satellites are considered reliable and red markers indicate that the satellites are identified as outliers.

In this section, the results of the T-RAIM algorithm are discussed. In Figure 3, the reliability flag determined by the T-RAIM algorithm is displayed for all satellites, with the GPS data on the left and the Galileo data on the right. A green marker signifies that a satellite is deemed reliable and can be included in the navigation solution, whereas a red marker indicates that a satellite is identified as a blunder and is therefore excluded from the navigation solution. The figure reveals that the algorithm only excludes satellites during the activation of ramps, accurately identifying those affected. Throughout the rest of the test, no satellites are excluded.

**Figure 4:**
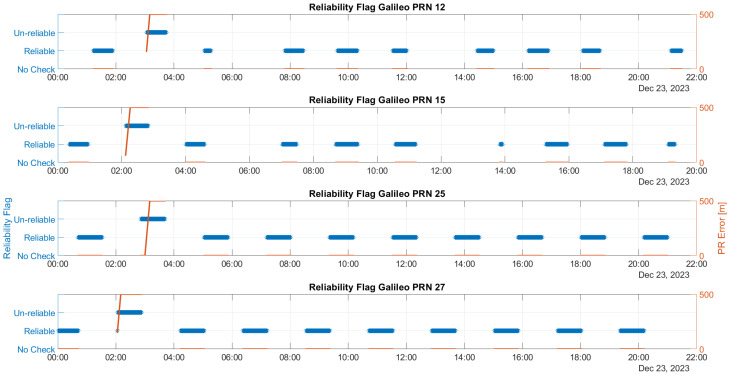
Time evolution of the reliability flag for Galileo satellites affected by ramp errors, with a dual y-axis displaying reliability flag values (blue, left) and ramp values (red, right).

**Figure 5:**
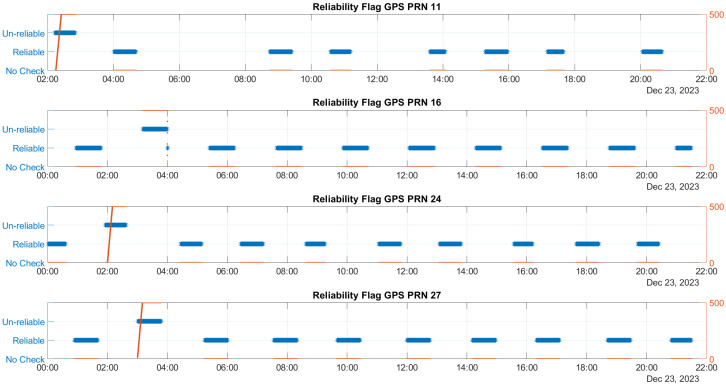
Time evolution of the reliability flag for GPS satellites affected by ramp errors, with a dual y-axis displaying reliability flag values (blue, left) and ramp values (red, right).

The time evolution of the reliability flag for Galileo and GPS satellites influenced by ramp errors is illustrated in Figure 4 and Figure 5, respectively. These figures feature a dual y-axis: the left side displays the reliability flag values in blue, while the right side shows the ramp values in red.

From the figures, it can be noted that the algorithm classifies satellites as unreliable only when faults are activated. Detection occurs at different ramp values, which are influenced by the SLOPE values specific to each satellite. The satellite that produces the largest estimation error due to its bias error is the most challenging to detect and is correlated with the highest SLOPE value. This indicates that the detection is dependent on the individual characteristics of each satellite, particularly in relation to their SLOPE values. This is primarily a qualitative assessment. While a detailed correlation analysis between SLOPE parameters and detection performance would be essential, it falls beyond the scope of this study and is reserved for future work.

**Figure 6:**
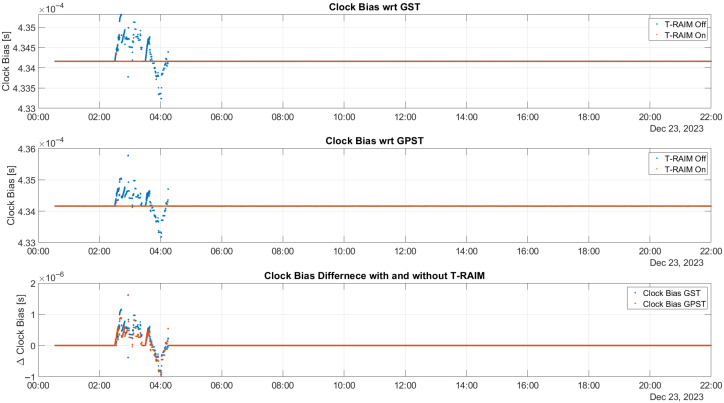
Clock bias estimates over time relative to GST (top) and GPST (middle), with blue dots for estimates without T-RAIM and red markers for estimates with T-RAIM.

The effect of activating T-RAIM on clock bias estimation is illustrated in Figure 6. In this study, the receiver clock bias is estimated for the multi-constellation considering two clock biases: one with respect to GPS time (GPST) and the other one with respect to Galileo System Time (GST). The top box of the figure shows the time evolution of the clock bias relative to GST, while the middle box considers the case for GPST. In both plots, the blue dots represent estimated values without the activation of T-RAIM, and the red markers denote the estimated values when T-RAIM is activated. In the lower box, the differences between the estimated values with and without T-RAIM are shown for GST (blue dots) and GPST (red dots). The figure shows that the estimation of clock biases is strongly affected during the activation of ramp errors for both the GPS and Galileo cases. In contrast, when T-RAIM is activated, the clock biases maintain their stability. Throughout the test, the difference between clock biases estimated with and without T-RAIM remains at zero, except for the periods when ramp errors are active. During these times, the difference can reach 1 microsecond, which can cause interruptions in the LEO-PNT service. This highlights the effectiveness of T-RAIM in maintaining clock bias stability and mitigating the impact of ramp errors on LEO-PNT services.

### 4.2. P2OD with and Without T-RAIM

This section examines orbit reconstruction and clock steering derived from the P2OD algorithm, comparing its performance in stand-alone mode versus integration with the T-RAIM algorithm.

**Figure 7:**
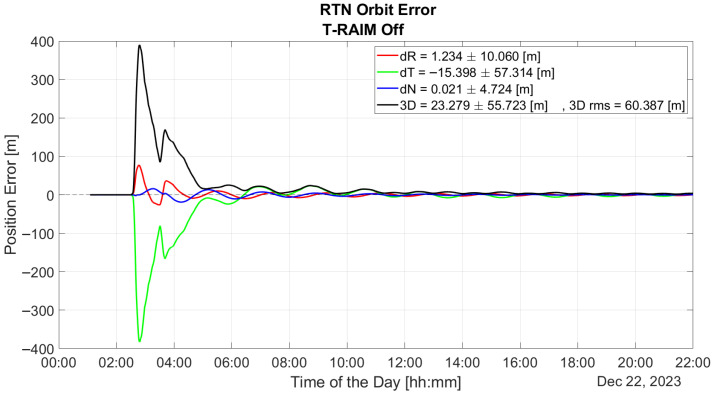
Time evolution of the P2OD orbit error in RTN reference frame without T-RAIM.

Figure 7 illustrates the time evolution of orbit error over the entire test duration, calculated in the Radial, Transverse, and Normal (RTN) reference frame with T-RAIM disabled. The figure highlights the P2OD solution’s divergence when one or more PR errors are present. The first hour is excluded from all P2OD plots to bypass the initial convergence phase, focusing instead on nominal operating conditions of the LEO-PNT payload.

**Figure 8:**
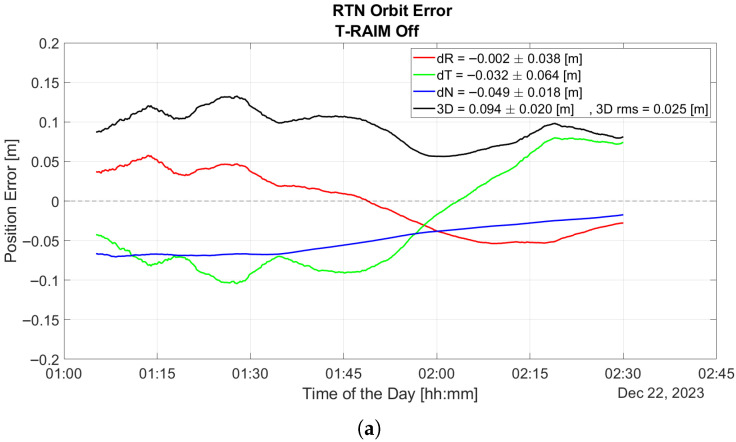

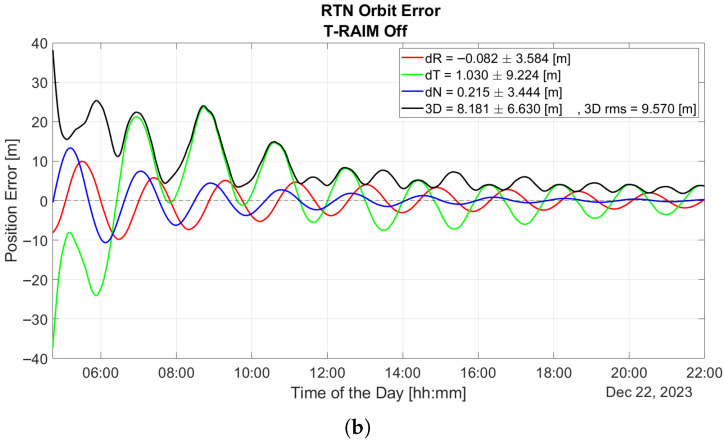
P2OD performance details before (**a**) and after (**b**) the fault.

The plot underscores the P2OD algorithm’s sensitivity to GNSS faults. Notably, before any fault is introduced (as shown in Figure 8a), P2OD achieves decimeter-level accuracy, meeting the requirements for nominal ephemeris generation during LEO-PNT operations. Without any quality check a measurement outlier results in a significant overshoot of the performance and a subsequent transient lasting several hours where errors remain well above the meter level, as shown in Figure 8b.

**Figure 9:**
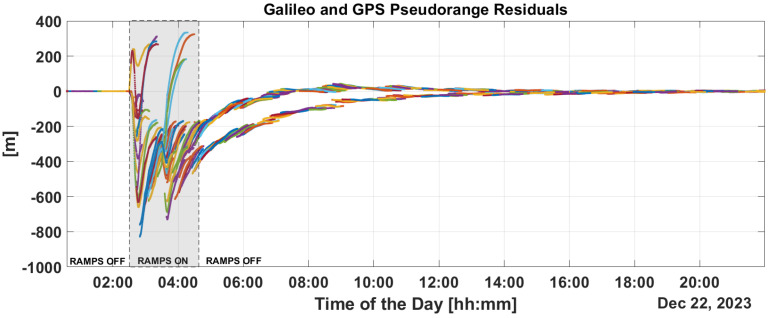
PR residuals without and with T-RAIM activation.

**Figure 10:**
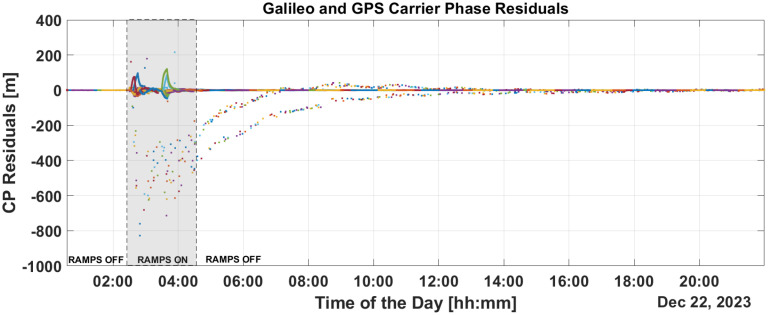
CPresiduals without and with T-RAIM activation.

Figure 9 and Figure 10 show the time evolution of PR and CP residuals during fault injection. These figures highlight the rapid spread of errors from the fault across all residuals. Both the PR and CP residuals exceed values of 500 m. The filter fails to mitigate the impact of these outliers, and even after the PR ramps are disabled, significant errors persist in the residuals which only diminish after a period of eight hours.

**Figure 11:**
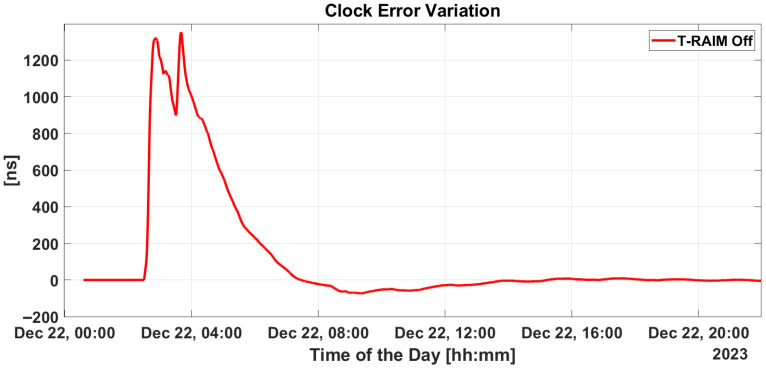
Clock bias variation without T-RAIM activation.

Figure 11 depicts the variation in estimated clock bias with and without T-RAIM. Initially, the difference is zero at the start of the test but rises to as much as 1200 ns during ramp activation. This discrepancy only returns to zero after several hours. A similar plot for Single-Point Positioning is presented in the lower box of Figure 6, where the difference returns to zero almost immediately after the ramp is turned off, avoiding the prolonged re-convergence period required by the filter.

**Figure 12:**
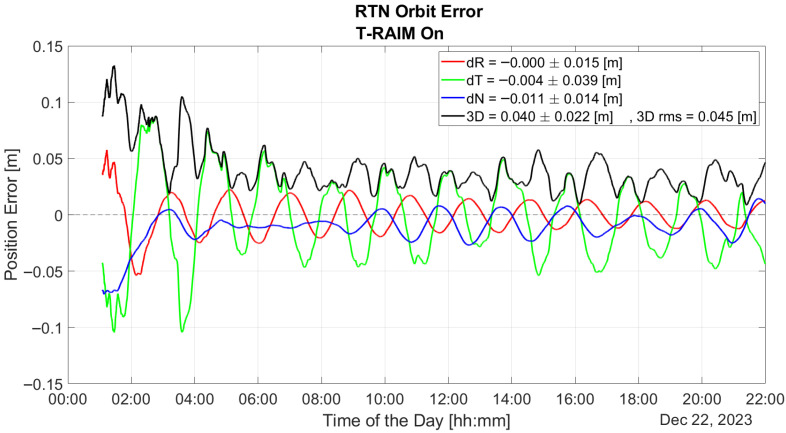
P2OD orbit error in RTN reference frame with T-RAIM activation.

The benefits of our lightweight approach, based on pre-filtering and T-RAIM, are demonstrated in Figure 12. Pre-detection enables the P2OD filter to use blunder-free measurements and provide orbit estimation at the decimeter level for the whole duration of the test.

**Figure 13:**
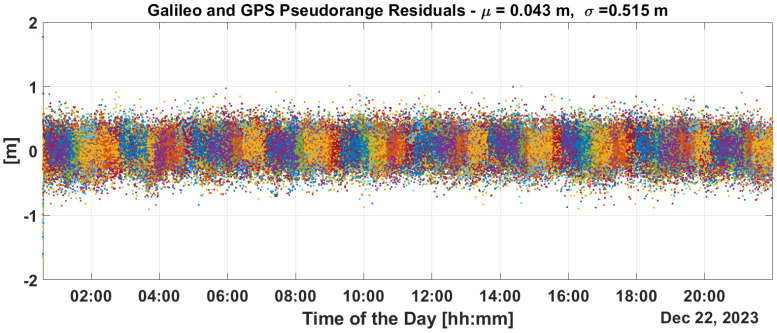
PR residuals with T-RAIM activation.

**Figure 14:**
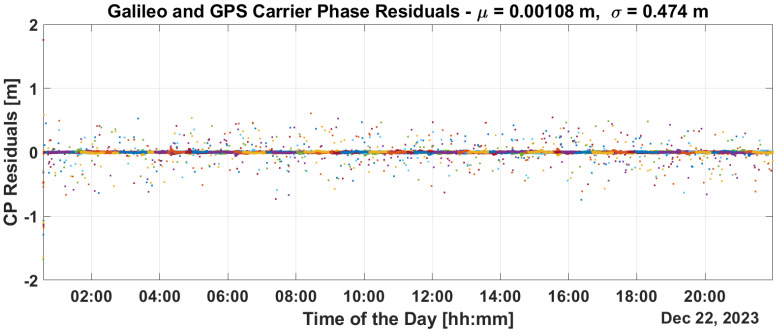
CP residuals with T-RAIM activation.

This is confirmed in the PR and CP residuals analysis provided in Figure 13 and Figure 14. From the figures, it can be noted that PR and CP residuals remains at the decimeter and millimeter levels, respectively.

**Figure 15:**
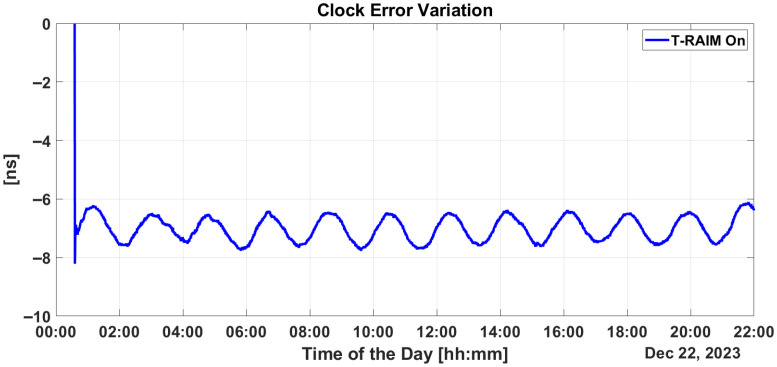
Clock bias variation with T-RAIM activation.

Regarding synchronization performance with the introduction of the proposed integrity layer, an estimation of clock bias error is provided in Figure 15. From the figure, P2OD’s ability to maintain clock error variation stably within +/−1 ns throughout the scenario clearly emerges, confirming its capability to limit the corresponding ranging generation in LEO-PNT.

**Figure 16:**
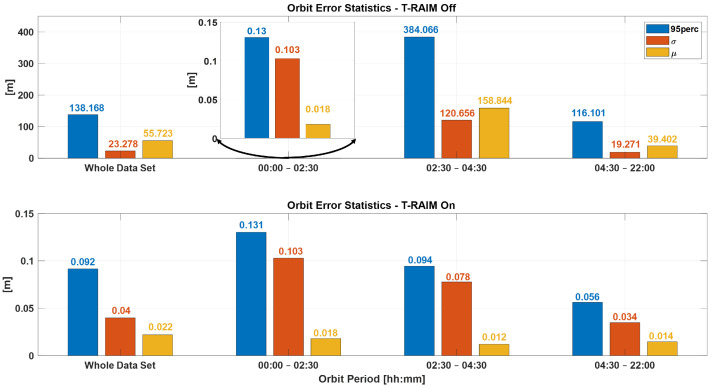
Statistical error parameters before during and after the pseudorange ramps considering the solutions with T-RAIM (lower box) and without T-RAIM (upper box).

The comparison between performance with and without T-RAIM is summarized in Figure 16 which provides errors statistics before, during, and after the fault. It is important to note that the T-RAIM filter performs transparently before a fault occurs, with no fault detection, and the filter’s evolution remains unchanged. During the faulty phase, missed detection drastically degrades performance, whereas pre-filtering enables to maintain the errors in the order of ten cm. A point to highlight is that excluding a few satellites does not degrade performance, which remains within the accuracy bounds expected for LEO-PNT service provision. This is a result of the dynamic filter’s ability to better manage a reduced number of measurements rather than faulty ones. This confirms that redundancy, while fundamental, is not a significant issue for P2OD and LEO-PNT operations compared with fault injection, which can be detrimental to service continuity.

## 5. Discussion

Implementing some baseline integrity techniques is essential to preventing the failure modes shown in the previous section. Figure 9 and Figure 10 show a critical effect, where the error rapidly spreads across all residuals. Consequently, a global test at the P2OD level is recommended for the LEO-PNT system to detect and inform users when the system is not working under nominal conditions. When the screening of the residuals indicates an issue, LEO-PNT must be considered faulty because signals follow an incorrect clock reference, generating large errors in the LEO measurements, and the LEO signal should be flagged as unhealthy. This is evident from the “clock fault” variation (computed between nominal and faulty P2OD clock values). In an autonomous multi-tier system, the unhealthy condition may last longer than the MEO fault itself due to the long recovery time needed by the filter (as shown in Figure 11), though ground intervention could limit the outage to the effective two hours. To achieve real autonomy, on-board isolation and mitigation techniques are necessary to improve service continuity but are challenging to implement within P2OD due to limited on-board resources. Our approach based on T-RAIM provides the first step in this direction, with a light and simple implementation to cope with stringent spaceborne receiver requirements. The pre-detection function enables the P2OD filter to proceed nominally maintaining decimeter-level orbit estimation. The success is further confirmed by the residuals’ behavior and tight clock control. The T-RAIM filter performs transparently before a fault occurs, with no fault detection. Crucially, excluding a few satellites does not degrade performance below accuracy bounds. This is a result of the dynamic filter’s ability to better manage a reduced number of measurements rather than faulty ones, confirming that redundancy, while fundamental, is not a significant issue for P2OD and LEO-PNT operations compared with fault injection, which is detrimental to service continuity. While the approach demonstrates that excluding a few satellites does not degrade performance below accuracy bounds, it relies on the dynamic filter’s ability to manage fewer measurements effectively. This reliance on redundancy may not adequately address scenarios with multiple simultaneous faults, where measurement availability is severely compromised. This aspect remains an open topic for future research activities.

## 6. Conclusions

This paper proposes and validates the use of T-RAIM algorithms in highly dynamic LEO environments, where rapid changes in satellite visibility are common. This study developed and tested an open-loop integration architecture that combines T-RAIM with P2OD. This integration is designed to maintain accurate orbit determination and timing even in the presence of faults, without the need for P2OD process restarts, which could disrupt LEO-PNT services. The approach is demonstrated using the JRC test environment. The results show that the integrated system effectively mitigates the impact of pseudorange ramp errors, maintaining stable clock bias and preventing error propagation into critical positioning and timing domains for end users. Without T-RAIM, the P2OD algorithm experiences substantial divergence in orbit error during pseudorange faults, leading to performance overshoots and transient errors that exceed meter levels. However, with T-RAIM activation, the orbit reconstruction maintains decimeter-level accuracy even under fault conditions, ensuring that the system meets the nominal requirements for LEO-PNT operations. In terms of residuals, the pseudorange and Carrier-Phase residuals without T-RAIM show considerable deviations during fault injection (up to hundreds of meters), but with T-RAIM, these residuals remain confined within decimeter and millimeter levels, respectively.

The proposed architecture is capable of managing the computational demands while accommodating the limited resources typical of spaceborne receivers, thus offering a robust solution for LEO-PNT applications. These findings underscore the importance of incorporating integrity monitoring capabilities into spaceborne receivers to enhance the robustness and reliability of emerging LEO-PNT systems.

## Figures and Tables

**Table 1:** P2OD dynamic model settings.

Item	Description
Force models	
Earth gravity field	EIGEN-6c4, deg = 50, order = 50
Third-body perturbations	Point Mass model for Sun and Moon
Solid Earth tides	Compliant with IERS 2003
Relativity	Post-Newtonian corrections
Solar radiation pressure	Cannon-ball model, Conical Earth shadow
Atmospheric drag	Cannon-ball model, NRLMSISE-00 density model
Empirical accelerations	First-order Gauss–Markov process in RTN directions
Observation model	
Measurement type	Ionosphere-free combination of dual-frequency pseudorange and Carrier-Phase GNSS observations
Phase wind-up	Corrected
Numerical integration	Runge–Kutta–Hull (2)4
Estimation	
Type	Extended Kalman Filter
State parameters	Position, velocity, drag, and SRP coefficients;
	empirical accelerations; receiver clock error; inter-system bias;
	Carrier-Phase ambiguities

**Table 2:** Pseudorange error implementation.

System	SV	Start Time	Ramp-Up Time	Max Error	Hold Time
	ID	[hh:mm]	[hh:mm]	[m]	[hh:mm]
Galileo	27	02:30	00:10	500	01:00
	15	02:36	00:10	500	01:00
	25	03:30	00:10	500	01:00
	12	03:30	00:10	500	01:00
GPS	24	02:30	00:10	500	01:00
	11	02:45	00:10	500	01:00
	27	03:30	00:10	500	01:00
	16	03:30	00:10	500	01:00

## Data Availability

The raw data supporting the conclusions of this article will be made available by the authors on request.

## Abbreviations

The following abbreviations are used in this manuscript:

ARP	Approximate Radial-error Protection
EUSPA	European Union Agency for the Space Programme
EC	European Commission
EMSL	European Microwave Signature Laboratory
ESA	European Space Agency
FB	Forward/Backward
FDE	Fault Detection and Exclusion
GNSS	Global Navigation Satellite System
GNSST	GNSS Time
GT	Global Test
HAS	High-Accuracy Service
HIL	Hardware-in-the-loop
HPL	Horizontal Protection Level
HW	Hardware
IRIS2	Infrastructure for Resilience, Interconnectivity and Security by Satellite
JRC	Joint Research Centre of the European Commission
JRC-ISSVTB	JRC Integrated SSV Test Bench
KPI	Key Performance Indicator
LEO	Low Earth Orbit
LEO-PNT	Low Earth Orbit Position, Navigation, and Timing
LT	Local Test
MEO	Medium Earth Orbit
ODTS	Orbit Determination and Time Synchronization
P2OD	Precise Real-Time On-board Orbit Determination
PNT	Position, Navigation, and Timing
PPP	Precise Point Positioning
PPS	Pulse Per Second
RAIM	Receiver Autonomous Integrity Monitoring
SPP	Single-Point Positioning
SSV	Space Service Volume
SSV-MS	SSV Mission Simulator
SV	Space Vehicle
SW	Software
T-RAIM	Timing Receiver Autonomous Integrity Monitoring
TAL	Time Alarm Limit
UniPD	University of Padua
WATP	Weighted Approximated Time Protected
WTS	Weighted Timing Slope
WLS	Weighted Least Squares
